# Production of IL1-beta by ovarian cancer cells induces mesothelial cell beta1-integrin expression facilitating peritoneal dissemination

**DOI:** 10.1186/1757-2215-5-7

**Published:** 2012-02-01

**Authors:** Takafumi Watanabe, Toshihiro Hashimoto, Takashi Sugino, Shu Soeda, Hiroshi Nishiyama, Yutaka Morimura, Hidekazu Yamada, Steve Goodison, Keiya Fujimori

**Affiliations:** 1 Department of Obstetrics and Gynecology, Fukushima Medical University School of Medicine, Fukushima, Japan; 2 Department of Gynecology, Tsuboi Cancer Center Hospital, Koriyama, Japan; 3 Department of Pathology, Fukushima Medical University School of Medicine, Fukushima, Japan; 4 Cancer Research Institute, M. D. Anderson Cancer Center Orlando, Orlando, FL, USA

**Keywords:** ovarian cancer, peritoneal dissemination, IL-1β, β1 integrin, mesothelial cell

## Abstract

**Background:**

A crucial step in the metastatic spread of ovarian cancer (OC) is the adhesion and implantation of tumor cells to the peritoneal mesothelium. In order to study this step in the cascade, we derived a pro-metastatic human ovarian carcinoma cell line (MFOC3) from the non-metastatic FOC3 line.

**Methods:**

Molecular profiling of the isogeneic lines identified differentially expressed genes, and investigation for a role in dissemination for specific factors was achieved by development of a co-culture adhesion assay utilizing monolayers of human mesothelial cells.

**Results:**

After murine intraperitoneal inoculation, the FOC3 cell line formed no metastases, but the MFOC3 subline formed metastases in > 80% of SCID mice. MFOC3 cells also adhered 2-3 times more avidly to mesothelial monolayers. This adhesion was inhibited by neutralizing antibodies to IL-1β and enhanced by recombinant IL-1β (*p *< 0.01). IL-1β induced mesothelial cell β1-integrin, and an antibody to this subunit also inhibited the adhesion of MFOC3 to mesothelial cells *in vitro *and significantly reduced metastases *in vivo*. Immunohistochemical analysis of a cohort of 96 ovarian cancer cases showed that negative IL-1β expression was significantly associated with an improved overall survival rate.

**Conclusions:**

These results suggest that a IL-1β/β1-integrin axis plays a role in ovarian tumor cell adhesion to mesothelia, a crucial step in ovarian cancer dissemination.

## Background

Ovarian cancer (OC) is the most lethal gynecologic malignancy in industrialized countries. The overall 5-year survival rate of ovarian cancer patients is 30% to 50%, largely due to the fact that the majority of these patients are diagnosed at an advanced stage (III or IV) of disease at initial diagnosis [1]. Substantial advances in the treatment of primary OC have occurred, but patient morbidity and mortality remain high due to metastatic dissemination [2]. Ovarian tumor cells primarily disseminate by shedding into the peritoneal cavity where they can implant on to the mesothelium that covers the omentum and bowel surface [3]. In order for the tumor cells to establish secondary foci and invade the underlying stroma, they need to adhere to and interact with the peritoneal mesothelial cells. This is a crucial step in OC progression and is a possible target for chemotherapeutic intervention, yet few studies have focused on this interaction. Identifying crucial factors involved in the crosstalk between the tumor cells and the mesothelial microenvironment will not only improve our understanding of the disease but will ultimately enable us to provide better patient care.

Details of the mechanisms involved in OC cell adherence to mesothelium are unclear, but the dynamics of this interaction appear to be relatively rapid, in the order of minutes [4,5]. Potential molecular interactions include tumor cell binding to extracellular matrix proteins such as collagens type I and IV, laminin, and fibronectin via integrins, and binding to hyaluronan expressed on the surface of human peritoneal mesothelial cells via CD44 [5-7]. The secretion of proteolytic enzymes, cytokines, and growth factors by both tumor cells and mesothelium will contribute to the regulation of adherence, survival and further dissemination. Recent studies have demonstrated that c-Met overexpression is a prognostic factor in ovarian cancer and that targeting c-Met in vivo inhibits peritoneal dissemination and invasion through an α5 β1 integrin-dependent mechanism [8].

In order to facilitate the study of ovarian tumor cell-mesothelial cell interactions, in this study we established a novel human ovarian cancer xenograft model, and a reproducible OC adherence assay for the investigation of the interaction and behavior of a highly metastatic OC cell line (MFOC3). Differential gene expression analysis was employed to identify genes with potential mechanistic influence, and one of these, interleukin-1beta (IL-1β) was found to be pivotal for increased adherence of the ovarian cell line to human mesothelial cells. Immunohistochemical evaluation of ovarian tumor tissues revealed a significant correlation between with IL-1β expression and overall survival. The derivation of this ovarian tumor xenograft model provides a powerful experimental system for the controlled evaluation of molecular mechanisms involved in ovarian carcinoma dissemination and metastasis.

## Methods

### Ovarian cancer cell culture

The human ovarian cancer cell line, FOC3 was kindly provided by Dr. T. Fukuda (Fukushima Medical University, Fukushima, Japan) [9]. Ovarian cancer (OC) cell lines were maintained in Dulbecco's modified Eagle's medium (DMEM) in the presence of 10% fetal bovine serum (FBS) and 50 U penicillin-streptomycin at 37°C in a humidified atmosphere of 5% CO2.

### Establishment of an intraperitoneal human ovarian cancer xenograft model

Exponentially growing cells were harvested with trypsin-EDTA, washed and resuspended in FBS-free DMEM. For subcutaneous (s.q.) inoculation, 1 × 10^7 ^FOC3 cells in 0.2 ml of DMEM/50% Matrigel (BD Bioscience, San Jose, CA) were injected s.q. into the left inguinal region of 6 week-old female severe combined immunodeficiency (SCID) female mice (Nihon Clea, Tokyo, Japan). FOC3 and MFOC3 cells were injected into 10 and 16 animals respectively. When the primary xenograft tumor reached a diameter of 1 cm, the tumor was removed, and minced with scissors aseptically, and seeded into a petri dish. After several passages, the surviving culture was designated MFOC3. A total of 1 × 10^7 ^FOC3 and MFOC3 cells in 0.5 ml PBS were injected intraperitoneally (i.p.) into SCID female mice. All mice were sacrificed 13 weeks after the injection and visible tumor, mesenterium, peritoneum, omentum and diaphragm were removed, fixed in 20% buffered formalin and processed for hematoxylin and eosin (H&E) staining. Frequency of peritoneal metastasis between FOC3 and MFOC3 were compared by the χ2 test (StatView, SAS Institute, Cary, NC, USA). *p *< 0.05 was considered to be statistically significant. This experiment was approved by the Institutional Animal Care and Use Committee of Fukushima Medical University (Ref# KO3129).

### Primary culture of Mesothelial cells

Human mesothelial cells (MCs) were isolated from the peritoneum of patients who were undergoing surgery for benign conditions at Fukushima Medical University School of Medicine and Tsuboi hospital. Written informed consent was obtained from all patients as per IRB approval from the Fukushima Medical University School of Medicine, and all documentation is retained within patient files. The procedure was based on techniques described in previous articles^7,9-15^. Briefly, MCs were collected by scrubbing the peritoneum with a cold knife. The MCs were cultured using DMEM contained 10% FBS and 50 U penicillin-streptomycin at 37°C in a humidified atmosphere of 5% CO2. The MC's were used in experiments at passage 2-5.

### Microarray analysis

Difference mRNA transcript expression between FOC3 and MFOC3 was investigated using the Human Cancer CHIP microarray, version 2.1 (Takara, Shiga, Japan) containing probes to 557 human genes reportedly associated with cancer. Total RNA was isolated from culture cells using TRIZOL (GIBCO, Grand Island, NY), and mRNA was further purified using an oligotexTM-dT30 'Super-mRNA Purification kit' (Takara). cDNA labeled with Cy3-dUTP or Cy5-dUTP was generated from 1 ug of FOC3 and MFOC3 mRNA using Takara RNA Fluorescence Labeling Core Kit (Takara), and 50 pg of lambda A was labeled as an internal control. Cy3 and Cy5-labeled probe were co-hybridized to the microarrays in hybridization solution (6 × SSC, 0.2% SDS, 5 × Denhardt's solution, carrier DNA) at 65°C over night, and the microarray slide processed as previously described (Nagata, et al, 2003). The Cy3 and Cy5 fluorescence intensities were captured with a 428 array scanner (Affymetrix, Santa Clara, CA) with separate measurement of the sample intensities for Cy3 and Cy5. The intensity of each hybridization signal was evaluated photometrically using Imagene software (BioDiscovery, Marina Del Rey, CA) and normalized to the average signal of a housekeeping gene, GAPDH. The Cy3/Cy5 ratio for each sample was calculated by averaging the spots, and the cutoff value for each expression level was calculated according to the background noise. For this cutoff, we used an expression level of above 100 where the fluctuation was less than a critical value (1.0) because other genes (those with low expression) are embedded in the background noise. Upregulated and downregulated genes in the MFOC3 cells were defined as those having a Cy3/Cy5 signal ratio of > 3.0 and < 0.50, respectively.

### RT-PCR

We confirmed the differential expression of the IL-1β gene using RT-PCR. 2 ug of total RNA was reverse-transcribed using a first strand cDNA synthesis kit (Invitrogen, Carlsbad, CA). 1 μl of the first strand cDNA preparation was added to the PCR reaction. The primers for IL-1β were as described by McAlindon et. al. [10]. Oligonucleotide IL-1β primers; 5'ATGGCAGAAGTACCTAAGCTCGC-3' and 5'-TTGACTGAAGTGGTACGTTAAACACA-3' (PCR product 802 bp). GAPDH primers; 5'-GCACAGTCAAGGCAGAGAGAAC-3' and 5'-CTTATGACCACTGTCCACGC-3' (PCR product 420 bp). PCR was performed using TAKARA™ Ex Taq (Takara). PCR for IL-1β was performed with 30 cycle of denaturation for 30 seconds at 94°C, annealing for 30 seconds at 62°C, and elongation for 1 minute at 72°C. PCR for GAPDH was performed with 20 cycle of denaturation for 30 seconds at 94°C, annealing for 30 seconds at 56°C, and elongation for 1 min at 72°C. PCR products were electropohoresed on a 1.5% agarose gel and visualized using ethidium bromide staining.

### Western blot analysis

In order to confirm the differential expression of IL-1 β in the cell lines, we performed Western blot analysis. Freshly cultured OC cells were lysed in a CellLytic™-MT reagent (Sigma, St. Louis, MO), and protein concentration of lysates were quantified by the Bradford method using Bio-Rad protein assay (Bio-Rad, Hercules, CA). Cell lysates (20 ug) were electrophoresed on BIO-RAD ready gels 4-20% Tris-HCL (Bio-Rad) and electrically transferred to a PDVF membrane (Bio-Rad). The membrane was blocked with 1xTBS, 5% nonfat dry milk, 0.05% Tween20 and incubated overnight at 4°C with 2 ug/ml mouse anti-human IL-1β antibody (R&D Systems, Abinghdon, UK), and 1 hr at room temperature with 1:2000 dilution of anti-mouse IgG conjugated HRP (Amersham Pharmacia Biotech, Tokyo, Japan). As a loading control, horseradish peroxidase-conjugated polyclonal antibody to β-actin (1:2000) (Santa Cruz, Santa Cruz, CA) was applied as described for primary antibodies above. Immunoreactive bands were visualized using ECL Western blot detection system (Amersham Pharmacia Biotech) following manufacturer's protocol.

### GFP transfection of OC cell lines

Transfection was performed using SuperFect^® ^Transfection Reagent (Qiagen, Tokyo, Japan) according to the manufacture's protocol, with minor modification. Briefly, 2 × 10^5 ^of OC cells were placed in a 35 mm dish the day prior to transfection. 2 ug of the pEGFP-N1 plasmid (Clontech, Palo Alto, CA) was mixed with 12 μl of SuperFect^® ^reagent per dish in serum-free DMEM (final volume of 60 μl), and DNA-lipid complexes were formed by incubation for 10 min at room temperature. DNA-lipid complex was incubated with FOC3 and MFOC3 cells for 3 hours, cells were washed with PBS, and 2 ml DMEM with 10% FBS was added. Three days later, the cells were exposed to 500 ug/ml G418. The limiting dilution method was used with medium containing G418 (500 ug/ml; Promega, Madison, WI) for generation of monoclonal cell lines. Single, GFP-positive cells in 96-well plates were detected using fluorescence microscopy (Olympus, Tokyo, Japan). To confirm the tumorigenicity of GFP labeled cells, these derivative lines were inoculated into SCID mice (as described above). In the absence of any co-inoculated cytokines, GFP labeled FOC3 (0/10 animals) and MFOC3 (8/10 animals) had very similar propensity to the unlabeled cell lines with respect to peritoneal tumorigenesis.

### In vitro adhesion assay

In order to compare the difference between FOC3 and MFOC3 adhesion to mesothelial cells (MCs), 1 × 10^5 ^GFP labeled FOC3 and MFOC3 cells in serum free DMEM were placed over mesothelial monolayers in an uncoated 24-well plate and incubated at 37°C for various periods. Students's *t*-test was performed as a test of significance (StatView, SAS Institute, Cary, NC). Data was presented as mean ± SD. *p *< 0.05 was considered to be statistically significant.

The effect of recombinant IL-1β on OC adhesion to mesothelium was achieved by adding different concentrations of recombinant IL-1β (R&D) in serum free DMEM incubated at 37°C for 30 minutes. To observe inhibition of IL-1β and integrin β1 on OC cell adhesion, 1 × 10^5 ^FOC3-GFP, MFOC3-GFP and FOC3-GFP containing 20 ng/ml recombinant IL-1β and neutralizing antibodies of IL-1β and β1 integrin (Chemicon, Temecula, CA) in serum free DMEM were placed over mesothelial monolayers in an uncoated 24-well plate and incubated at 37°C for 60 minutes. Normal mouse IgG (Amersham Pharmacia Biotech) was used as a negative control. Both antibodies were used at 10 ug/ml, since preliminary experiments demonstrated that this concentration produced maximum inhibition of OC cell binding to mesothelium. For all assessments, nonadherent cells were removed by washing twice with PBS after incubation. The adherent cells were fixed by 4% paraformaldehyde and counted at 100x under a fluorescence microscope. These data were analyzed by one-way analysis of variance with the Bonferroni/Dunn test (StatView). Data was presented as mean ± SD. *P *< 0.05 was considered to be statistically significant.

### In Vivo Assessment of Ovarian Cancer Peritoneal Metastasis

To determine the effect of IL-1β and integrin β1 antibody on ovarian cancer peritoneal metastasis *in vivo*, 1 × 10^7 ^MFOC3 cells with anti-IL-1β (10 ug/ml) and anti- β1 integrin (10 ug/ml) in 0.5 ml PBS were injected i.p. into SCID female mouse. Normal mouse IgG (10 ug/ml) was used as a negative control. All mice were sacrificed after 13 weeks, and tumors visible on the mesenterium, peritoneum and diaphragm were monitored under low-power magnification. Frequency of peritoneal metastasis between MFOC3 and MFOC3 with anti IL-1β or anti β1 integrin were compared by the χ2 test (StatView). *p *< 0.05 was considered to be statistically significant.

### Flow cytometry

Analysis of adhesion molecules in MCs was detected using Flow cytometry. At confluency, MCs were washed twice with fresh culture media and then incubated with 20 ng/ml recombinant IL-1β in serum free RPMI 1640 for 30 minutes. MCs were harvested using trypsin (0.05%) and EDTA (0.02%) and resuspended in PBS with 2% FBS. 100,000 cells treated with primary monoclonal antibodies for integrin α1 (Chemicon), integrin α2 (SouthernBiotec, Birmingham, AL), integrin α5 (Cymbus Biotechnology, Hampshire), integrin α6 (Chemicon), integrin αV (Chemicon) and CD44 (SouthernBiotec) at a dilution of 1:15, and integrin β1 (Chemicon) at a dilution 1:100, at 4°C for 30 minutes. Cells were washed twice in PBS with 2% FBS, and labeled with FITC-conjugated rabbit-anti-mouse Ig (Dako, Tokyo, Japan) at 4°C for 30 minutes. After two additional washes, the cells were analyzed using a flow cytometry (BD Bioscience, San Jose, CA). Statistical analysis was performed using the Students's *t*-test for paired data (StatView). Fluorescence intensities are presented as median ± SD absolute deviation in relation to control, which were set as 100%. *p *< 0.05 was considered to be statistically significant.

### Patient Population

Patients with a diagnosis of early stage (stages I and II) ovarian carcinoma were identified at Fukushima Medical University School of Medicine and Tsuboi hospital during a 10 year period from 1995 to 2004. The clinical stage was determined using FIGO criteria. A total of 96 patients had primary surgical cytoreductive surgery with no exposure to prior chemotherapy. The median age of the patients was 50.7 years (range 12-86). All patients gave informed consent before their inclusion in this study. Association between IL-1β expression and age, histology and ascites were assessed using χ2 test (StatView). *p *< 0.05 was considered to be statistically significant.

### Immunohistochemistry

Paraffin-embedded tissue sections were deparaffinized, and endogenous peroxidases were inactivated by a 15-min treatment with methanol containing 3% hydrogen peroxide. Sections were subjected to heat-induced antigen retrieval for 15 min at 100°C in 10 mM citrate buffer before addition of the primary antibody. After nonspecific binding was blocked with goat serum for 30 min, all slides were incubated overnight at 4°C for overnight with a 1:50 dilution of rabbit antihuman IL-1β antibody (Santa Cruz Biotechnology, Santa Cruz, CA). After incubation, an avidin-biotin-peroxidase reaction was performed using Histofine SAB-PO(R) kit (Nichirei, Tokyo, Japan) following manufacturer's protocol. The sections were subjected to diaminobenzidine for 10 min and counterstained with Mayer's hematoxylin.

IL-1β expression was estimated in a combined scoring system based on both the percentage of positive cells (negative = 0, < 10% = 1, 11-50% = 2, and 51-80% = 3, > 81% = 4) and the staining intensity (negative = 0, weak = 1, moderate = 2, and strong = 3). The final immunoreaction score (IRS) was obtained by multiplication of the % value of positive cells by the value for staining intensity. The IRS can range from 0 to 12, and the cases of 4 to 12 and 0 to 3 were defined as high and low expression of IL-1β respectively. Survival curves were calculated using the Kaplan-Meier method, and differences in survival were analyzed using the log-rank test (SPSS 12.0, SPSS Co., Tokyo, Japan). In all analyses, statistical significance was defined as a probability value less than 0.05.

## Results

### Selection of FOC3 cells with a high propensity for peritoneal metastasis

**Figure 1:**
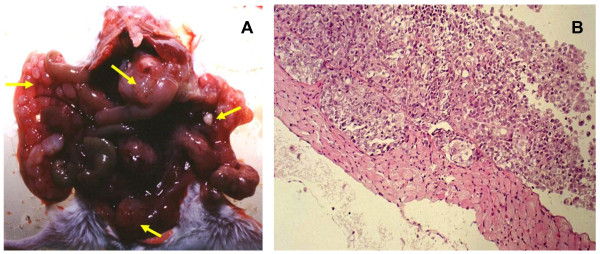
**Representative appearance of intraperitoneal (i.p.) tumor implants in SCID mice**. **A**. Macroscopic view of a peritoneal metastatic tumor after i.p. injection of MFOC3. Small round metastatic foci of extensive local growth (arrowhead) are present in the peritoneum, omentum and mesenterium. **B**. Microscopic view of a peritoneal metastatic tumor after i.p. injection of MFOC3. MFOC3 cells (arrowhead) grew on the peritoneum.

The human ovarian cancer cell line FOC3 was established from a human ovarian serous cystadenocarcinoma [9]. FOC3 does not exhibit metastatic potential when introduced into athymic mice by intraperitoneal implantation. As shown in other xenograft models, prolonged growth of a cancer cell line in a murine host can adapt, or select cells for more robust growth in the same host in a subsequent inoculation. In order to derive a more aggressive FOC3 cell population we inoculated 1 × 10^7 ^cells into the inguinal region of female SCID mice, the xenograft was recovered, disaggregated and cultured for several passages. Proliferation of the FOC3 and MFOC3 cell lines did not differ significantly in tissue culture. This sub-population, designated MFOC3, and the parental FOC3 population were injected intraperitoneally into SCID under the same conditions. At 13 weeks, FOC3 tumors were not discovered in any of ten mice. Conversely, peritoneal tumors were evident in 13 of 16 mice (81.3%) inoculated with MFOC3 cells (Table 1A). MFOC3 tumors were observed on the mesenterium, peritoneum, omentum, and diaphragm (Figures 1A and 1B). FOC3 cells form a monolayer sheet of polygonal cells with pavement-like arrangement in culture. In culture, no major distinct morphological differences between the parental FOC3 and the malignant MFOC3 cells were observed.

**Table 1:** Peritoneal Metastasis and Inhibition

A) Peritoneal metastasis after intraperitoneal transplantation of FOC3 and MFOC3		
Cell Line	Number of Mice	Number of Mice with Metastasis (%)
FOC3	10	0(0)
MFOC3	16	13 (81.3) *

		*FOC3 vs MFOC3: p < 0.01 (χ2 test)
**B) Inhibition of Peritoneal metastasis in MFOC3 cells by anti-IL-1**β **and anti- integrin β1**		
**Antibody**	**Number of Mice**	**Number of Mice with Metastasis (%)**

control	10	8 (80) a
IL-1β	10	7 (70) b
Integrin β1	10	2 (20) c

		*a vs b: non significant;*
		*a vs c: p < 0.01 (χ2 test)*

### Differentially gene expression associated with peritoneal metastasis

Gene expression profiles of FOC3 and MFOC3 cells were compared in order to identify genes that may be associated with the metastatic or non-metastatic cellular phenotype. mRNA samples were profiled using the Human Cancer CHIP microarray, which contains 557 human cancer-related genes.

**Figure 2:**
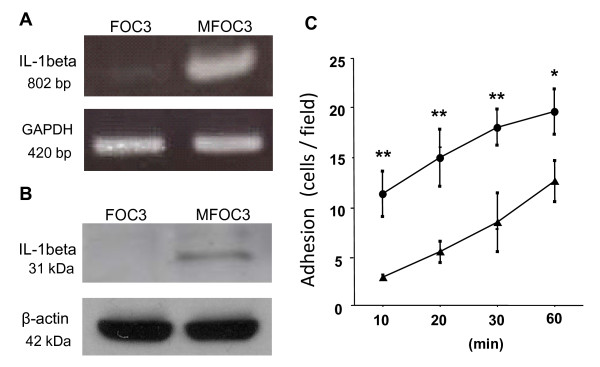
**Validation of IL-1β expression and cell adhesion assay**. **A**. RT-PCR analysis of IL-1beta was performed on FOC3 and MFOC3 mRNA using GAPDH as the internal control. **B**. Expression of IL-1β protein. Western blot analysis was performed on FOC3 and MFOC3 cell samples with anti- IL-1β, using β-actin as the loading control. **C**. Tumor cell adhesion assay. GFP-labeled FOC3 (▲) and MFOC3 (●) cells were added to the confluent mesothelial cell monolayer for 10, 20, 30 and 60 minutes and counted using fluorescent microscopy after washing away unattached cells. Each point represents the mean ± SD of at least three times separate experiments. * p < 0.05, ** p < 0.01.

The analysis revealed 41 up-regulated genes (> 3-fold change), and 37 down-regulated genes (< 0.50-fold change) in MFOC3 cells. Selected genes are listed in Table 2 and the full lists of differentially expressed genes are available on request. Genes up-regulated in MFOC3 included cell adhesion molecules (ADAM9, ITGA8, CTNNΒ1), proteolytic enzymes (PAI2), cytokines (IL1β, TNF), growth factors (FGF1, NRG, TGFΒ1) and apoptosis inhibitors (API1, API2). The expression level of PAI2 in MFOC3 was 25 times higher than that of FOC3. Genes in the keratin cytoskeleton family (KRT19, KRT14, KRT18 and KRT13) were consistently decreased in MFOC3. The expression level of KRT19 was 10 times lower in MFOC3 than in FOC3. Decreased expression of cell adhesion molecules (ITGB8, ITGA9), caspase 1 and an inhibitor of metastasis (NME1) were also observed in MFOC3 cells. The increased expression of interleukin-1beta (IL-1β) in metastatic MFOC3 cells was of particular interest to us because it has previously been implicated in cancer cell dissemination [11]. To validate the microarray profile result, we monitored the comparative levels of IL-1β using RT-PCR analysis. IL-1β mRNA transcript levels were confirmed to be significantly higher in MFOC3 cells (Figure 2A). The differential expression of IL-1β protein was confirmed to follow the same pattern using Western blot analysis (Figure 2B).

**Table 2:** Selected genes shown to be differentially expressed between Foc3 and MFOC3 cells by transcriptomic profiling.

*Downregulated in MFOC3*			
Gene Symbol	Gene Name	Fold Change	Function
KRT19	Keratin 19	0.10	cytoskeleton
KRT14	Keratin 14	0.13	cytoskeleton
ITGB8	Integrin beta 8	0.19	cell and matrix adhesion
KRT13	Keratin 18	0.24	cytoskeleton
KRT18	Keratin 13	0.30	cytoskeleton
CASP1	Caspase 1	0.33	IL1beta convertase
ITGA9	Integrin alpha 9	0.39	cell and matrix adhesion
ESR1	Estrogen receptor 1	0.41	hormone receptor
NME1	Non-metastatic protein 1	0.49	inhibitor of metastasis
***Upregulated in MFOC3***			
**Gene Symbol**	**Gene Name**	**Fold Change**	**Function**

PAI2	Plasminogen activator inhibitor type II	25.56	proteolytic enzyme
IL1B	Interleukin 1beta	11.11	cytokine
TNF	Tumor necrosis factor	7.88	cytokine
API2	Apoptosis inhibitor 2	7.44	inhibition of apoptosis
TGFB1	Transforming growth factor	5.30	cell adhesion regulation
PLAU	Plasminogen activator, urokinase	4.50	proteolytic enzyme
FGFR1	Fibroblast factor receptor 1	4.34	growth factor receptor
API1	Apoptosis inhibitor 1	4.26	inhibition of apoptosis
ITGA8	Integrin alpha 8	3.95	cell and matrix adhesion
LAMB1	Laminin, beta1	3.88	extracellular matrix
FGF1	Fibroblast growth factor 1	3.79	growth factor
SDC1	Syndecan 1	3.19	membrane protein
CTNNB1	Catenin, beta1	3.03	cell adhesion

### Cell adhesion is influenced by tumor cell IL-1β and mesothelial cell β1 integrin

A reproducible assay was designed to test the adhesion of FOC3 and MFOC3 cells to human mesothelial cells (MCs). MCs were maintained in culture as a monolayer for 2-5 passages. GFP labeled FOC3 and MFOC3 cells were allowed to bind to the MCs for 10 min, 20 min, 30 min and 60 min. Adherent cells remaining after washing were counted. Adhesion of OCs increased with time (Figure 2C). MFOC3 cells adhered more readily to MCs than FOC3. The increased adherence rate observed was in the order of 2-3 times greater, and remained constant up to 30 minutes (Figure 2C). For all subsequent adhesion assays, the 30 min time point was used.

**Figure 3:**
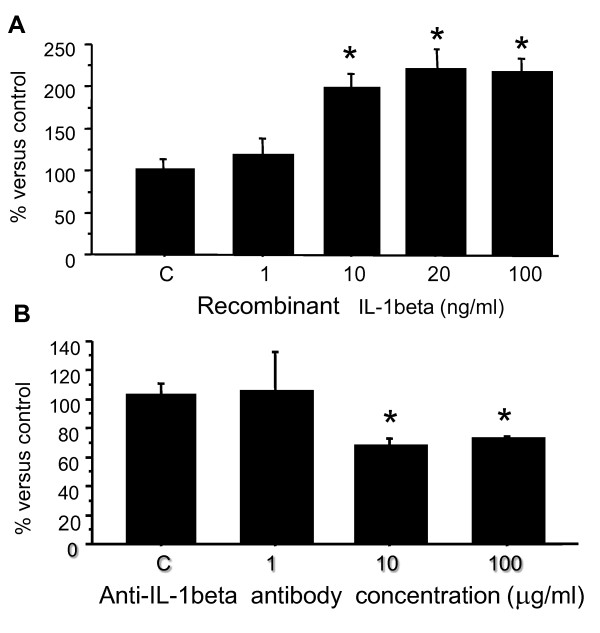
**A. FOC3 cell adhesion to mesothelial cells after pre-incubation with 1, 10, 20 or 100 ng/ml recombinant IL-1β**. * p < 0.01 **B**. Inhibition of MFOC3 adhesion to mesothelial cells by 1, 10 or 100 ug/ml anti-IL-1β. Control (C) experiment was performed in the presence of 100 ug/ml mouse IgG. Data are expressed as the mean ± SD of three times separate experiments. * p < 0.05.

**Figure 4:**
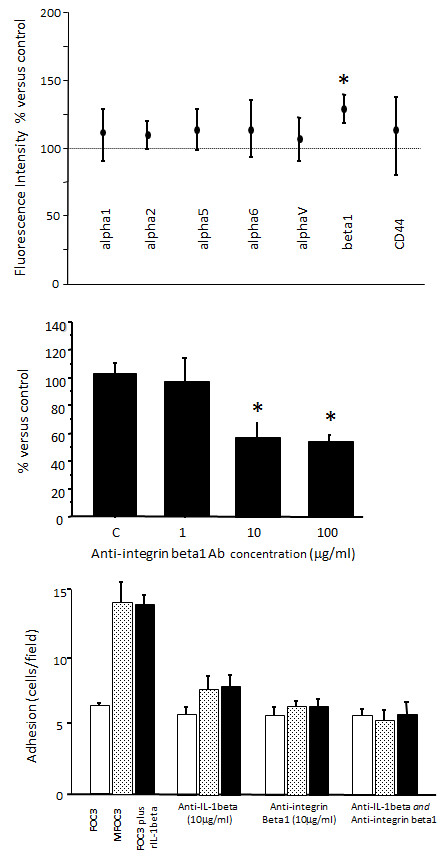
**A. Expression of cell adhesion molecules on mesothelial cells (MCs)**. MCs were exposed to IL-1β (20 ng/ml) for 30 minutes, and expression of a panel of adhesion molecules was assessed by flow cytometry. Untreated control was set as 100%. Results are presented as median values ± median absolute deviation in relation to control. Data are representative of five experiments. * p < 0.01. **B**. Inhibition of MFOC3 adhesion to mesothelial cells by 1, 10 or 100 ug/ml anti-β1 integrin * p < 0.05. Control (C) experiment was performed in the presence of 100 ug/ml mouse IgG. **C**. Summary of the effects of anti-IL-1β (10 ug/ml) and β1 integrin (10 ug/ml) on the binding to mesothelial cells of FOC3 and MFOC3, and FOC3 with recombinant IL-1β (20 ng/ml). Open bars represent FOC3; shaded bars, MFOC3; solid bars, FOC3 with recombinant IL-1β. Data are expressed as the mean ± SD of three separate experiments.

Preincubation of MCs with recombinant IL-1β caused a significant increase in FOC3 adhesion to (Figure 3A). This effect was dose-dependent. Significant stimulation was already achieved at 10 ng/ml, and maximum stimulation was achieved with the addition of 20 ng/ml IL-1β (*p *< 0.01) in the adhesion assay. The augmented adhesion at this concentration was at least double that of adhesion achieved in the absence of recombinant IL-1β. Furthermore, inclusion of a neutralizing IL-1β antibody in the assay significantly decreased MFOC3 adhesion to MCs in a concentration-dependent manner (Figure 3B), with maximal inhibition at ~65% of control using 10 μg/ml antibody. These findings indicate that IL-1β plays a pivotal role in the adhesion of FOC3 cells to MCs. To investigate the specific effect of IL-1β on MCs, we performed flow cytometry to monitor the expression of a panel of cell adhesion molecules. Treatment of MCs with IL-1β (20 ng/ml for 30 minutes) resulted in a significant increase in the expression of β1 integrin (Figure 4A). The median level of β1 integrin was increased by 21.5% (P < 0.01) in the presence of IL1β. Treatment of MCs with IL-1β had no significant effect on the expression of α-1, 2, 5, 6, and V integrins, or the CD44 (Figure 4A).

Inclusion of a neutralizing anti-β1 integrin antibody in the adhesion assay significantly inhibited MFOC3 cell adhesion to MCs in a dose-dependent fashion. Reduction of adhesion to approximately 60% of control was observed at 10 and 100 μg/ml (Figure 4B). Figure 3C depicts a graphical summary of the effects of recombinant IL-1β, anti-IL-1β antibody and anti-β1 integrin antibody on FOC3 and MFOC3 cell adhesion. The adhesion of MFOC3 to MCs was greater than that of FOC3, however, the adhesiveness of FOC3 to MCs was increased to a level similar to that of MFOC3 by recombinant IL-1β (Figure 4C). The adhesion of both MFOC3 and FOC3 cells to MCs were inhibited by treatment with neutralizing anti- IL-1β and anti-β1 integrin antibodies to an equivalent extent, but the two antibodies did not have a synergistic effect. Through immunochemical staining and flow cytometry the well-characterized β1 integrin ligands fibronectin and laminin were found to be present equally in FOC3 and MFOC3 cells (data not shown).

### Effect of IL-1β in a murine metastasis model

To determine the effect of IL-1β and β1 integrin antibodies on ovarian cancer peritoneal metastasis *in vivo*, 1 × 10^7 ^MFOC3 cells with anti-IL-1β (10 ug/ml) and anti- β1 integrin (10 ug/ml) were administered together in 0.5 ml PBS were injected i.p. into SCID female mouse. Normal mouse IgG (10 ug/ml) was used as a negative control. Peritoneal metastasis resulting from i.p. inoculation of MFOC3 was observed in 8 of 10 mice (80%) (Table 1B). MFOC3 metastases were also observed in 7 of 10 (70%) mice treated with anti IL-1β. In contrast, MFOC3 produced peritoneal metastasis in only 2 of 10 (20%) mice when mice were treated with anti β1 integrin antibodies (Table 2). Treatment with anti-β1 integrin resulted in a significant decrease (p < 0.01) in the number of mice with metastases compared to control.

### Relationship between IL-1β expression and prognosis in early-stage ovarian cancer patients

**Figure 5:**
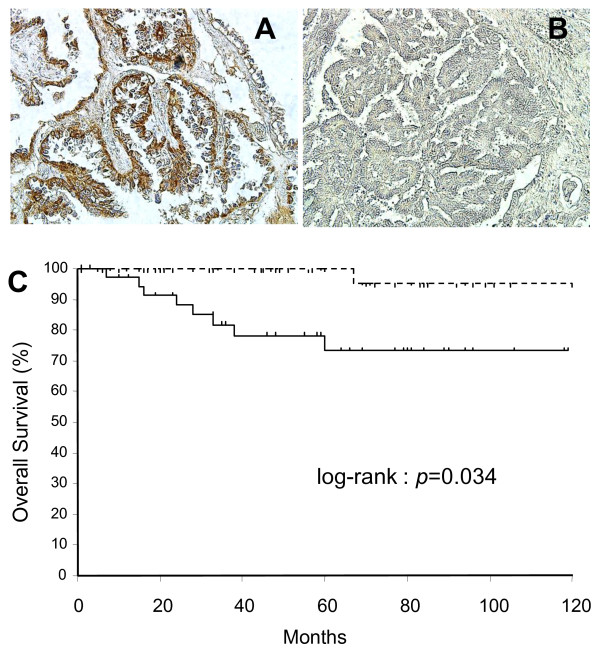
**IL-1β expression in ovarian tissues**. **A**. High expression in a section of ovarian carcinoma. Immunoreactions appeared in both the nucleus and cytoplasm of tumor cells (x200). **B**. Low/no expression in a section of ovarian tumor cells (x200). **C**. Kaplan-Meier analysis of overall survival of 96 patients with ovarian cancer with IL-1β high expression (solid line; n = 31) versus low/no expression (dashed line; n = 65).

We investigated the expression of IL-1β in archival tissues obtained from 96 early-stage ovarian cancer cases. The histological summary of the cases is described in Table 3; serous (25 of 96; 26.0%), mucinous (24 of 96; 25.0%), endometrioid (26 of 96; 27%), and clear cell (21 of 96; 21.9%). Immunohistochemical analyses revealed that many cases of OC were positive for IL-1β expression (Figures 5A,B). Expression of IL-1β was scored as high or positive (IRS score 4-12) and low or negative (IRS score 0-3). High expression of IL-1β was rated as 20.0% in serous, 37.5% in mucinous, 30.7% in endometrioid, and 42.8% in clear cell cases (Supplemental S1). High expression of IL-1β was observed in 32.3% (31/96) of all early-stage OCs examined. There was no significant association between histological type and IL-1β expression (*p *= 0.372), although the IL-1β high expression rate in clear and mucinous tumors was greater than that observed in serous and endometrioid tumors. Patients older than 50 years, and patients with ascites were more likely to be positive for IL-1β (37.2 versus 26.7% and 36.7 versus 28.8 respectively), but this did not reach statistical significance. Kaplan Meier survival plots of overall survival revealed that 9 of 31 (29%) patients whose tumors expressed IL-1β died from disease (most often from peritoneal metastases) within the follow-up period (5 years), compared to only 1 of 65 (1.6%) patients whose tumors did not express IL-1β (Figure 5C). IL-1β expression was significantly associated with overall survival in early-stage OC cases (*p *= 0.034).

**Table 3:** Association of IL-1β expression and clinicopathological variables

	Number of patients	IL-1β high expression (%)	IL-1β low expression (%)	P- value *(χ2 test)*
Ovarian cancer (stage 1+2)	96	31 (32.2)	65 (67.8)	
Age (years)				
< 50	45	12 (26.7)	31 (73.2)	
> 51	51	19 (37.2)	34 (62.8)	0.471
Histological type				
Serous	25	5 (20.0)	20 (80)	
Mucinous	24	9 (37.5)	15 (62.5)	
Endometroid	26	8 (30.7)	18 (69.3)	
Clear cell	21	9 (42.8)	12 (57.1)	0.372
Ascites				
Negative	52	15 (28.8)	37 (71.2)	
Positive	54	16 (36.7)	28 (63.2)	0.432

## Discussion

Despite improvements in surgery and treatment of ovarian cancer, the long-term survival of patients with this disease is limited due to the intraperitoneal (i.p.) spread of tumor cells from the primary tumor mass. A crucial step in the establishment of secondary foci is the adherence of disseminated tumor cells to the mesothelial surfaces of the peritoneum. Thus, a potentially efficacious adjuvant therapy approach could involve the i.p. administration of agents that target tumor cell and mesothelial cell interactions. To better understand the pivotal processes, we developed an *in vitro *cell adhesion model in order to identify key molecules and to test their effect on phenotype. Findings from the model were subsequently confirmed in human tissue specimens.

By 'conditioning' FOC3 cells through immune-compromised mice [12,13] we derived the MFOC3 sub-line by recovery from an advanced FOC3 xenograft tumor formed in SCID mice. Unlike FOC3 cells, inoculation of the MFOC3 subline resulted in multiple tumors across the peritoneal cavity in multiple experiments. These isogeneic cell line populations constitute a valuable model for the elucidation of mechanisms involved in peritoneal metastasis of ovarian cancer cells. It is to be noted that our molecular profiling was restricted to 557 genes and so cannot be considered comprehensive, but of particular interest from our differential gene expression analyses was the confirmed increased expression of interleukin-1beta (IL-1β) in metastatic MFOC3 cells. IL-1β has previously been implicated in peritoneal mesothelium integrity [14] and in cancer cell dissemination [11,15]. Development of an *in vitro *cell adhesion assay using human mesothelial cells (MCs) enabled the testing of a role for IL-1β in OC/MC cell adhesion. Previous studies, have performed similar *in vitro *adhesion assays using isolated human mesothelial cells [16,17], but here we monitored intact adherent cells accurately by tracking GFP-labeled FOC3 and MFOC3 cells. In these assays, MFOC3 cells were observed to adhere up to 3 times more avidly than the parental FOC3 cell population, and a dose-dependent increase in FOC3 adhesion after preincubation of MCs with IL-1β was observed. The specificity of this effect was confirmed by neutralization with an IL-1β antibody and reduction of MFOC3 adhesion to MCs in a dose-dependent manner. These findings indicate that IL-1β plays a pivotal role in the adhesion of FOC3 cells to MCs. A role for IL-1β in cell adhesion has been reported in different model systems. For example, IL-1β promoted the adherence of human colorectal carcinoma cell lines and melanoma cell lines to endothelial cells in a dose-dependent manner [18,19].

To monitor the effects of IL-1β treatment on MCs we measured the expression of a panel of adhesion molecules. No differences were observed in the MC expression of CD44, or 5 of 6 integrins monitored by flow cytometry, but an increase in the expression of MC β1 integrin was evident. Inclusion of a neutralizing anti- β1 integrin antibody in our *in vitro *cell adhesion assay significantly inhibited MFOC3 cell adhesion to MCs in a dose-dependent fashion. In combination, these analyses show that the increased adhesion of MFOC3 to MCs is influenced by the release of IL-1β released from the ovarian tumor cells, and one consequence of this is the induction of cell surface β1 integrin on MCs. The interference of tumor cell adhesion with neutralizing antibodies was also tested *in vivo*. MFOC3 metastasis rates of ~80% were not significantly changed in mice treated with anti- IL-1β antibodies, explained by high rates of antibody clearance or stability in vivo, or sequestration of antibodies by murine cytokines or other factors, however, peritoneal metastasis was observed in only 20% of mice when treated with anti- β1 integrin antibodies. Coupled with the presence of ligands laminin and fibronectin on FOC3 and MFOC3 cells, this confirms a role for the β1 integrin subunit in MFOC3 metastasis to the peritoneal cavity.

In order to link the model findings to events in human disease, we investigated the expression of IL-1β in archival tumor tissues obtained from a cohort of 96 early-stage ovarian cancer cases. Immunohistochemical analyses revealed no significant association between histological type and IL-1β expression level, but the IL-1β expression rate was greater in clear and mucinous tumors than that observed in serous and endometrioid tumors, and patients with ascites were more likely to be positive for IL1β. Most importantly, overall survival analysis revealed that 29% of patients whose tumors expressed IL-1β died from the disease within a 5-year follow-up period. In contrast, only 1.6% of patients whose tumors did not express IL-1β died within this same period. These results suggest IL-1β might be associated with tumors of a more aggressive phenotype, and interference with its production or action may be a promising target for ovarian cancer therapy.

A number of studies have focused on identifying targets that may prove clinically useful in the management of ovarian cancer patients. Targets under development include TRAIL, BCL-2 family, EGFR and VEGF, and clinical trials evaluating agents that inhibit VEGF and EGFR signaling pathways in ovarian cancer have been performed [20,21]. Interestingly, there is a link between IL1b and VEGF. IL-1β triggers the release of pre-existing, sequestered VEGF from the extracellular matrix, that then homes to the endothelial compartment where it induces endothelial cell proliferation [11]. So IL-1β is upstream of VEGF in the angiogenic pathway and therefore plays a key role in the expansion of the vascular compartment during tumor progression. VEGF actions in enhancing ovarian tumor metastasis are also thought to be mediated through crosstalk with integrin cell adhesion receptors [22]. Previous reports have shown a role for β1 integrin in the adhesion of OCs to mesothelia, but the hypothesis tested has invariably been that it is the integrins on the OC cell surface that mediate the interaction with mesothelial cells [5,15]. Our study shows that IL-1β released from the tumor cells induces β1 integrins on the MCs and that axis mediates cell adhesion between the two cell types. Thus, the IL-1β/integrin axis may not only advance initial cell adhesion of ovarian tumor cells to the peritoneum, but trigger a cascade of events that favor the establishment of dispersed secondary tumors. Perturbation of this axis at any stage of disease may have significant benefit.

The peritoneal mesothelium is a highly specialized layer of epithelial cells that covers the entire surface of the abdominal cavity. It serves as a protective anatomical barrier, and as a low friction, non-adhesive interface allowing the movement of abdominal organs. Cytokines present in inflammatory or malignant peritoneal effusions may significantly affect the function and the structure of the peritoneal mesothelial cell lining [23]. The cytokine milieu can induce an 'activated' mesothelium which, in turn, results in the secretion of a variety of factors, including IL-6, IL-8, VEGF [24,25], and IL-1β [26,27]. In the case where the tumor cell secretes its own IL-1β, then the phenomenon can occur without injury, or in an enhanced way at sites already undergoing remodeling.

It is likely that IL-1B plays multiple roles in progression and/or metastasis in ovarian cancer. It has been demonstrated that IL-1β can cause major morphological changes and loss of mesothelium integrity, characterized by cellular retraction and wide-spread exposure of the submesothelial ECM [14]. The disruption of the mesothelial barrier may be important for the progression of neoplastic disease [28]. Once ovarian carcinoma cells present in the peritoneal fluid adhere to mesothelial cells, the cancer cells may migrate through this layer, invade the local organs, and spread to distant sites. This idea is supported by an *in vivo *model study in which damage to the mesothelium caused by a pneumoperitoneum resulted in peritoneal tumor spread and a significant decrease in animal survival [29]. Other groups have studied the effect of peritoneal wounding on tumor cell adhesion. The adhesion of CC531, Caco2 and HT29 colon carcinoma cell lines to an autologous monolayer of rat mesothelial cells [30,31] pre-incubated with factors released after surgical trauma (IL-1β and EGF) resulted in at least 60% more cell adhesion in a dose-dependent manner. Additional blocking experiments with anti-IL-1β resulted in significant inhibition of the tumor cell adhesion in the model, but they did not test the effect *in vivo*. Both IL-1β and TNF has been shown to regulate ovarian cancer in nude mouse xenograft models [32]. Intraperitoneal recombinant IL-1β administration led to a dose dependent replacement of peritoneal ascitic tumour with solid tumours attached to the peritoneum, and high doses had an anti-tumor effect. Recombinant human TNF also promoted tumor implantation in the same xenograft models, but had no anti-tumor effect. There has been literature reporting an association between inherited polymorphisms in the IL-1β gene and various cancers, including cervical, bladder and breast, but the results remain ambiguous [33,34].

Thus, anti- IL-1β therapy may act on several levels; reduce tumor cell adhesion, maintain peritoneal integrity, and ameliorate peritoneal tumor cell induced inflammatory responses that may exacerbate adhesion and establishment of secondary tumors. Furthermore, because the frequency of IL-1β expression in human ovarian cancer specimens was strongly associated with overall survival, it is possible that IL-1β may also serve as a biomarker for prognosis or as a monitoring biomarker for therapeutic response. Additional tumor samples need to be evaluated to investigate the value of IL-1β as a biomarker with clinical utility.

## Conclusions

Cancers originating from organs in the peritoneal cavity (*e.g*., ovarian, pancreatic, colorectal, gastric and hepatic) present treatment difficulties precisely because of the occurrence of peritoneal metastases. A logical treatment option is intraperitoneal therapy after surgical removal of the primary tumor. The clinical utility of this approach has been hampered by toxicity issues, in part due to the lack of products designed specifically for intraperitoneal therapy. Targeted immunotherapy with monoclonal antibodies (mAbs) is a promising strategy in this context. A mAb could selectively target tumor cells or the mesothelium if we can identify those targets that are biologically essential to peritoneal dissemination. Therapeutic mAbs against vascular endothelial growth factor (VEGF) or its receptor, and human epidermal growth factor receptors [20,21] are being developed for ovarian cancer therapy. Clinical trials evaluating such agents, alone and in combination with chemotherapy, are urgently needed. It is no doubt likely that more than one subset of cell adhesion molecules will play a role in overall efficiency of secondary tumor establishment, but our studies show that a paracrine IL-1β/β1 integrin axis may play an important role in the early events of ovarian tumor cell metastasis and deserves further investigation in this context.

## List of abbreviations

OC: ovarian cancer; IL: interleukin; FBS: fetal bovine serum; U: units; RT-PCR: reverse-transcriptase polymerase chain reaction; GFP: green fluorescent protein; MCs: mesothelial cells.

## Competing interests

The authors declare that they have no competing interests.

## Authors' contributions

TW participated in all aspects of the study, from design to clinical and laboratory performance, and manuscript writing. TH established MFOC3 and participated in the cDNA microarray analyses. TS aided in conception, design, and experimentation, particularly immunohistochemistry. SS performed cell culture and adhesion assays. HM performed cell culture and immunoassays. SG participated in design, data analysis and drafting of the manuscript. YM, HY and KF participated in design and analysis of clinical data. All authors have read and approved the manuscript.
